# Hepatitis C Virus Vaccine: Challenges and Prospects

**DOI:** 10.3390/vaccines8010090

**Published:** 2020-02-17

**Authors:** Joshua D. Duncan, Richard A. Urbanowicz, Alexander W. Tarr, Jonathan K. Ball

**Affiliations:** 1 School of Life Sciences, The University of Nottingham, Nottingham NG7 2UH, UK; richard.urbanowicz@nottingham.ac.uk (R.A.U.); alex.tarr@nottingham.ac.uk (A.W.T.); jonathan.ball@nottingham.ac.uk (J.K.B.); 2 NIHR Nottingham BRC, Nottingham University Hospitals NHS Trust and the University of Nottingham, Nottingham NG7 2UH, UK; 3 Nottingham Digestive Diseases Centre, School of Medicine, University of Nottingham, Nottingham NG7 2UH, UK

**Keywords:** hepatitis C virus, vaccines, neutralising antibodies, animal models, immune responses

## Abstract

The hepatitis C virus (HCV) causes both acute and chronic infection and continues to be a global problem despite advances in antiviral therapeutics. Current treatments fail to prevent reinfection and remain expensive, limiting their use to developed countries, and the asymptomatic nature of acute infection can result in individuals not receiving treatment and unknowingly spreading HCV. A prophylactic vaccine is therefore needed to control this virus. Thirty years since the discovery of HCV, there have been major gains in understanding the molecular biology and elucidating the immunological mechanisms that underpin spontaneous viral clearance, aiding rational vaccine design. This review discusses the challenges facing HCV vaccine design and the most recent and promising candidates being investigated.

## 1. Introduction

First discovered in 1989 [1], the hepatitis C virus (HCV) is a major global health burden. Current estimates of HCV prevalence state that approximately 1% of the world’s population are infected [2]. Chronic infection with HCV leads to cirrhosis of the liver and is associated with the development of hepatocellular carcinoma (HCC). Annually, 400,000 deaths are attributed to HCV and in the US, deaths from HCV have now overtaken those attributed to the human immunodeficiency virus (HIV) [3]. The extensive damage resulting from chronic infection makes this virus the leading cause for liver transplantation, a procedure that ultimately results in reinfection of the transplanted organ [4,5]. In recent years, the growing problem of HCV prompted the World Health Organisation to set a target to eliminate HCV as a public health burden by 2030. However, in the absence of a vaccine against HCV, this will prove challenging.

Therapeutic treatment of HCV has been vastly improved over the past decade due to the development of direct acting antivirals (DAAs). These compounds act as inhibitors of either the NS3/4a serine protease, NS5a or the NS5b RNA-dependent polymerase [6] and can achieve a 95% cure rate [7]. However, there are limitations to this strategy. Firstly, in order to treat an infection, a diagnosis must be made which may not occur in asymptomatic cases. It has recently been reported that the diagnosis rate in 2014 in the United Kingdom was estimated at capturing less than half of infected individuals [8]. Secondly, the cost of these therapeutics limits their use in developed countries, and all but excludes their use in low and middle-income countries with high HCV burdens. Thirdly, the ability of HCV to rapidly respond to selective pressures means that the emergence of DAA-resistant strains is a major risk [9,10]. Indeed, resistance-associated substitutions (RAS) have been detected in circulating HCV strains in treatment naive patients [11,12]. One such example of a RAS that has been reported in treatment-naïve patients is the C316 mutation which has been associated with resistance to non-nucleotide NS5B inhibitors such as Nesbuvir [13]. Another example is the S282T RAS which is associated with sofosbuvir resistance in vitro [14] although at the time of writing this polymorphism is not prevalent in clinical cohorts. The risk of DAA-resistance is mitigated by using combinational therapies of DAAs that target different HCV proteins.

The ability to effectively treat HCV infection has been a major achievement. However, there is growing evidence that HCV can leave lasting impacts upon its host post infection. For example, during infection extensive liver fibrosis can occur, which can persist for several years after viral clearance. Furthermore, persistent hyperfunctional CD8+ T cell phenotype has been reported following successful treatment with DAAs, suggesting continued immunological impairment [15]. There are also conflicting reports about the risk of hepatocellular carcinoma after virus clearance [16,17]. Understanding the long-term effects following virus clearance will take years more research as more data become available from successful cases. However, it is now apparent that therapeutics alone are unlikely to achieve the 2030 elimination target and thus a vaccine is urgently needed.

Modelling based on viral kinetics in reinfected individuals with pre-existing immunity has shown that transmission risk can be greatly reduced when an immune response occurs, despite detectable virus RNA titres [18]. This is of particular importance when considering vaccination of people who inject drugs, a high-risk group for HCV infection and reinfection. This suggests that a successful vaccine could be used to reduce viral titres rather than inducing sterilising immunity. This is a key point as it provides a realistic goal for HCV vaccine research.

## 2. Host Immune Responses to Hepatitis C Virus

Virus-host interactions determine the outcome of acute HCV infection. This interplay is complex and includes components of both the adaptive and innate host immune system. The most common scenario is progression to chronic infection. However, 25%–40% of individuals undergo spontaneous viral clearance (SVC) [19,20] within 12 months of infection [21,22]. Approximately 80% of these individuals will achieve SVC a second time following reinfection [23] with a marked decrease in viral RNA titres and reduced infection times compared to the first infection [23,24]. This indicates that initial infection can lead to the establishment of an immunological memory which can control subsequent HCV infection. This protective response consists of both humoral and cellular adaptive immune responses that do not result in sterilising immunity but prevent chronic infection. This phenomenon provides a benchmark for HCV vaccine research and thus is vital to elucidate.

### 2.1. Innate Immune Reponses

Innate immunity provides a first line of defence against viral infection. The liver presents a unique microenvironment that is enriched with cells that participate in this response, namely Kupffer cells, natural killer (NK) cells, hepatic stellate cells (HSCs) and macrophages. Initial innate responses are triggered by HCV-derived pathogen-associated molecular patterns (PAMPs) that are recognised by pattern recognition receptors (PRRs). Virion-associated PAMPs are the E1 and E2 glycoproteins [25,26], while intracellular detection of viral PAMPs, including viral proteins and RNA, is mediated by toll-like receptors (TLRs), nucleotide-binding oligomerisation domain (NOD)-like receptors (NLRs) and retinoic acid-inducible gene-I-like receptors (RLRs) [27,28]. Activation of signalling cascades downstream of these receptors results in the production of proinflammatory cytokines including interleukin-1β (IL-1β), IL-18 and type I and type III interferons (IFNs). These IFNs mediate upregulation of interferon stimulated genes (ISGs) in an autocrine and paracrine manner leading to an antiviral response in the liver [29,30]. However, the HCV viral proteins core, E2, NS3/4a and NS5a all impair the expression of ISGs through disruption of signalling cascades, allowing the virus to overcome the host innate response [31,32]. This results in an innate immune response that is incapable of clearing HCV.

Successful SVC is influenced by the outcome of innate immune responses. A major example is the association of single nucleotide polymorphisms (SNP) rs12979860 present in the type III IFN gene *IFNλ4* with SVC. In this case approximately 50% of individuals with a C/C genotype achieve SVC [33,34]. Additionally, NK inhibitory receptor, killer immunoglobulin receptor 2DL3 (KIR2DL3) and human leukocyte antigen C group 1 (HLA-C1) are associated with SVC [35] due to a reduced inhibition of cytotoxic NK activity [36,37].

### 2.2. Ceullar Immune Responses

Cellular immunity has long been associated with spontaneous HCV clearance [38] and is mediated through two main T cell subsets, the cytolytic CD8+ T cells and CD4+ helper T cells. CD8+ T cells destroy infected cells in a manner restricted by MHCI presented epitopes [39]. In contrast, recognition by CD4+ helper T cells is MHCII restricted and their role is to aid the function of CD8+ T cells and the establishment of T cell memory through the secretion of cytokines such as IFN-γ [40,41]. CD4+ T cells also aid B cell activation and a CD4+ T cell subset, follicular helper T cells (T_FH_) are required to establish a long-term antibody response [42,43]. HCV-specific T cells are detectable within the first 12 weeks of infection and target a broad range of HCV epitopes present on both structural and non-structural viral proteins [44]. During the progression to chronicity, the HCV-specific CD4+ T cells display an exhausted phenotype and the population collapses [45,46]. The decrease in CD4+ T cell function leads to a dysregulated CD8+ T cell response in which these cells become exhausted and dysfunctional with reports of continued IFN-γ secretion but an absence of cytolytic activity [47]. The reasons for this reduction in effective cellular responses are incompletely understood. The loss of functional HCV-specific T cells could be the result of host regulation of the immune system since persistent antigen stimulation could lead to the prolonged production of proinflammatory cytokines which in turn contributes to hepatic tissue damage.

The importance of T cells to SVC was first demonstrated in experimentally infected chimpanzee in which HCV persistence was observed in the absence of either a CD4+ or CD8+ T cell response [48,49]. Interestingly, when CD4+ T cells were depleted HCV persisted alongside functional CD8+ T cell responses. HCV-specific CD4+ T cells and CD8+ T cells are detectable during acute infection [45]. This provides strong evidence that the T cell responses have a major role in the outcome of HCV infection. HCV-specific CD4+ T cells are broadly targeting with the most common epitopes being found in the core, E2, NS3, NS4a, NS4b, NS5a and NS5b HCV proteins [44].

### 2.3. Humoral Immune Responses

Neutralising antibodies (nAbs) in the context of HCV infection were first described by Farci et al. [50], although their role in spontaneous clearance was disputed for many years due to reports of cell mediated clearance in seronegative individuals [51,52,53], suggesting that nAbs are not essential to achieve SVC. However, analysis of sera from individuals who cleared HCV has shown the presence of nAbs and these are detectable at earlier time points compared to acute infections that proceed to chronicity and are subsequently lost following viral clearance [54] suggesting that a rapid, short-lived humoral response is required for clearance [55,56,57,58]. It has recently been shown that nAbs generated within the first 100 days of infection often have a narrow neutralising capacity directed towards the founder virus [58]. The selective pressure exerted by nAbs upon the circulating strains can also drive the evolution of HCV towards escape mutations that compromise viral fitness further aiding clearance of the infection [59,60]. The delayed appearance of cross-reactive nAb responses are apparent in chronically infected individuals as isolated sera can neutralise circulating strains from previous infection time points with greater potency than the current dominant virus [56] and cross-reactive nAbs have been isolated from chronically infected individuals [61,62,63,64]. Although these nAbs cannot clear the infection, they have been associated with reduced liver fibrosis [65] and patients that experience hypogammaglobulinemia have a more severe disease progression [66].

Further insight into the humoral response has been obtained through characterising nAbs derived from patients. Bailey et al. [67] sequenced cross-reactive nAbs isolated from two individuals that cleared HCV and showed these nAbs shared >90% similarity with germline heavy chain variable (VH) genes and >92% similarity with germline light chain gene sequences revealing that generation of cross-reactive nAbs required limited somatic hypermutation. A common feature of these nAbs was the *VH1-69* gene which is found in potent cross-reactive nAbs that target antigenic region 3 (AR3) and is also present in nAbs targeting HIV and Influenza [68,69,70,71]. Structural investigation has also shown that cross-neutralising activity is a result of long complementarity-determining region H3 (CDRH3), typically 18–22 residues, that forms a β-hairpin structure that is stabilized by a disulphide bond allowing for interaction with conserved E2 epitopes [72,73]. This level of insight into how potent nAbs work can inform rational design of B-cell immunogens to favour the production of these types of antibodies in the host.

## 3. Hepatitis C Virus Envelope Proteins as Vaccine Targets

**Figure 1:**
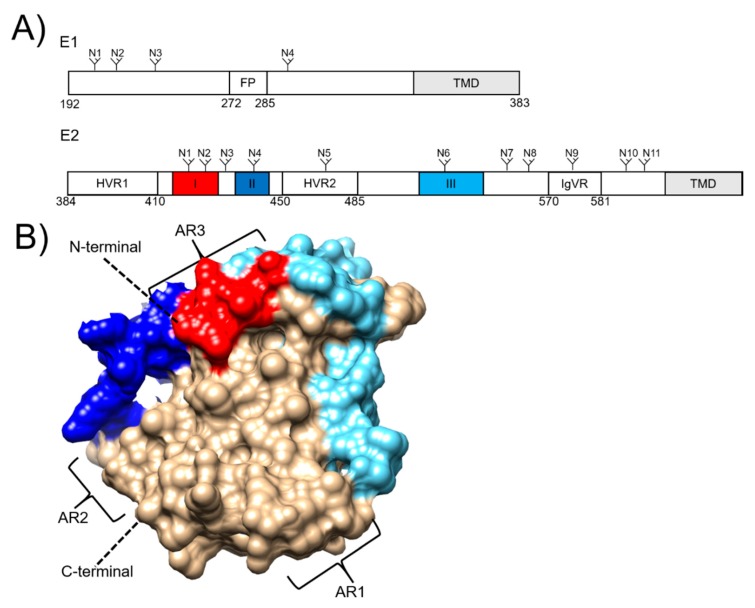
(**A**) Schematic diagrams of the hepatitis C virus envelope glycoproteins E1 and E2, showing N-linked glycosylation sites (N), transmembrane domains (TMDs), and the E1 fusion peptide (FP). E2 hypervariable regions (HVRs) 1 and 2, and the intergentypic variable region (IgVR) are also depicted. Linear epitopes I, II and III are highlighted in red, dark blue and light blue, respectively. (**B**) E2 structure (PDB: 6MEH). Linear epitopes I, II and III are highlighted in the corresponding schematic. Antigenic regions (ARs) are also shown.

The HCV envelope glycoproteins, E1 and E2, are located at residues 192–746 of the polyprotein and are the targets of the humoral immune response, making them an attractive vaccine target [74] (Figure 1A). Both E1 and E2 are type I transmembrane proteins that form an intracellular non-covalent heterodimer that form higher order covalent structures on the mature virion [75,76]. The E2 ectodomain contains an immunoglobulin-like β-sandwich that is flanked by α-helices which form a front and back layer [77,78]. There are also regions that exhibit high levels of variability which are referred to as hypervariable regions (HVRs) 1 and 2, and a third intergenotypic variable region (IgVR; Figure 1A). The E1 protein is smaller, more conserved than E2 and less well characterized with partial crystal structures resolved for fragments encompassing residues 192-271 [79] and 314–324 [80].

The E1E2 heterodimer mediates entry into the hepatocytes through interactions with four essential host receptors, CD81 [81,82], scavenger receptor B1 (SRB1) [83], claudin [84] and occludin [85,86]. The first step in viral entry is the interaction between SRB1 and the HVR1 (Figure 1A) located at the N-terminus of E2 which induces a conformational change that exposes the conserved E2 core region and the CD81 binding loop (residues 519–535) [77]. The interaction between E2 and CD81 results in recruitment of claudin to CD81 which leads to clathrin-mediated endocytosis [87]. Membrane fusion occurs in low pH endosomes, which is thought to induce a conformational change in the E1E2 heterodimer [88]. This leads to membrane fusion, possibly mediated by E1 via the action of a putative fusion peptide located at residues 272–285 (Figure 1A) [89].

Antibody-mediated neutralisation of HCV is achieved through targeting the E1E2 heterodimer on the surface of the virus. To date, most characterized nAbs target the E2 protein. The are several linear and discontinuous regions of E2 that are targeted by Abs and these have varying nomenclature, being referred to as ARs1-5 [69], Epitopes I-III (Figure 1) [90], or domains A-E [91]. Importantly, AR4 and 5 are reliant on the presence of the E1E2 heterodimer for binding [69]. The epitope I, II and AR3 regions form the neutralising face of E2 which is targeted by some of the most potent cross-reactive nAbs described to date [92] arguing for their inclusion in a nAb-eliciting vaccine.

## 4. Challenges to Hepatitis C Virus Vaccine Design

### 4.1. Genetic Diversity

As a species, HCV exhibits extensive genetic diversity is driven by a mutation rate in the order of 10^−4^ substitutions per site of the genome [93,94]. Mutations are acquired through the activity of the error prone NS5b RNA-dependent RNA polymerase and this coupled with high levels of virus production and selective pressures exerted by the host immune response has driven the diversification of HCV. There are currently eight genotypes (Gt1-8) reported which are defined by 30% difference in nucleotide sequence [95]. Gt1 HCV isolates have the highest prevalence, accounting for 49% of diagnosed cases globally followed by Gt3 accounting for 17.9% of cases [96], and are more prevalent in developed countries [96]. In contrast, Gt4 and Gt5 isolates are more prevalent in lower income countries in Africa and the Middle Eastern regions [96]. Genotypes are further classified into subtypes of which there are currently 90 confirmed groups. These exhibit a 15% variation in nucleotide sequence [95]. Gt1-7 contain multiple subtypes. However, the recently reported Gt8 group currently contains a single subtype isolated from four individuals [97]. Rapid evolution of HCV during the course of an infection leads to the establishment of a heterogeneous population [98]. The diversification of this population is driven by host immune selective pressure and the degree to which variation occurs in acute infection correlates with a progression to chronicity [99].

The extent of genetic diversity gives rise to genotype-specific immune responses. The E1E2 sequence shows the greatest level of variation as a result of the selective pressure exerted by the host immune response [100], leading to humoral responses that can have reduced heterologous neutralising activity [101]. However, cross-reactive nAbs that are capable of targeting isolates from different genotypes have been described in several studies [102], thus highlighting that this challenge can be overcome. Epitope variability also leads to genotype-specific cellular responses for example Luxenburger et al. [103] have recently shown CD8+ T cells from individuals chronically infected with a Gt4 HCV isolate failed to recognize Gt1 derived epitopes. Limited intergenotypic cross-reactivity of Gt3 HCV-specific T cells has also been reported in patients that successfully cleared Gt3 infections [104]. Limited cross-reactive immune responses to distantly related HCV isolates will be a key challenge to produce an effective vaccine

### 4.2. Evading the Host Adaptive Immune Response

The ability of HCV to establish a chronic infection highlights the efficiency in which this virus can subvert the host immune response. This is achieved through multiple strategies. Firstly, immunodominant epitopes such as the HVR1 of E2 elicit a response of non-neutralising antibodies. Currently it is thought that HVR1 acts as a shield of the more conserved epitopes in the E2 core that contain CD81 binding residues, since removal of HVR1 increases virus susceptibility to neutralisation [105,106]. Evidence also shows that HVR1 induces greater homologous nAbs and deletion induces broader heterologous nAbs [107].

Epitope masking is also achieved through the glycosylation of envelope proteins to form a glycan shield [108]. This mechanism diminishes the binding of nAbs and is used by other viruses such as HIV and Influenza although by comparison, HCV exhibits a reduced variability in the position of each glycan group. The E1 and E2 proteins contain 4 and 11 N-linked glycosylation sites which are highly conserved across different genotypes (Figure 1A) [109]. Deletion of E2 glycosylation sites increases HCV susceptibility to nAbs showing that glycosylation acts via steric hinderance to nAbs [109,110]. Interestingly, removal of E2 glycan sites or expression of this protein in systems that use smaller, less complex glycan groups enhance the immunogenicity compared to mammalian expressed E2 [111,112,113]. Another mechanism in which nAb resistance can be mediated through glycosylation is glycan shift. This arises through non-synonymous mutations that result in the deletion of a glycosylation site and the appearance of a new glycosylation site in a different position in the protein. Pantua et al. [114] reported this phenomenon after incubating cell cultured HCV (HCVcc) with the murine nAb, AP33 which targets residues within the E2 epitope I region [115]. After 5 days of incubation with AP33, escape mutants could be detected which contained either N417S or N417T residue variations coupled with a new glycosylation site at position 415 [114].

The evasion of nAbs can also be attributed to the presence of host derived factors. One such factor is high-density lipoproteins which are present in human serum and has been shown to increase HCV infectivity via SR-BI interactions which in turn decreases the time available for nAbs to bind to their target [116,117]. Additionally, the presence of host derived apolipoprotein E on the mature HCV virion has been shown to have a role in epitope masking the E2 protein abrogating nAb activity [118]. Another evasion method is through the use of decoys. Recently it has been reported that in a HCVcc system, infected cells produce lipid droplets coated with the E2 protein [119]. This may also be a strategy to subvert the immune system by using these E2-coated droplets to interact with nAbs, thereby reducing the availability of nAbs to target E2 present on the surface of the virus particle. Finally, evasion from nAbs has recently been shown to be associated with resistance to antiviral interferon-induced transmembrane (IFITM) proteins [120] showing that the innate immune response can also be a driving force for nAb evasion.

### 4.3. Models for Hepatitis C Virus Infection

#### 4.3.1. In Vitro Models

Since the discovery of HCV, research was hampered by the lack of infection models both in vivo and in vitro. The use of models in vaccine research is essential for the assessment of sera and monoclonal antibodies arising from natural infection, experimental immunizations or vaccine trials. In the case of in vitro models, the key challenge was generating a cell culture-based system that could produce infectious HCV at titres suitable for further experimentation. This problem was solved with the discovery of two Gt2a isolates, JFH1 and J6 which produce high titres of infectious HCV from cultured Huh 7.5 cells [121]. Following this, it was found that intergenotypic recombinant viruses consisting of core-NS2 of an isolate of interest fused to NS3-NS5b of JFH-1 could produce viable chimeric viruses [121]. The developments in HCVcc techniques have allowed panels with representatives of the seven major genotypes to be set up [122,123,124]. Our group has recently described a method in which E1E2 patient derived sequences could be incorporated into chimeric HCVcc expression vectors [125,126], further aiding the ability to characterize isolates that have different neutralisation phenotypes.

Viral pseudotyping has also been used to study the entry mechanism of HCV. This approach utilizes the ability of retroviral gag/pol proteins to self-assemble into enveloped virus particles. Simultaneous expression of foreign viral envelope proteins in the same cells leads to the incorporation on to the surface of the retroviral particle whose entry properties are dictated by the envelope glycoprotein. Infectivity can be measured by the incorporation of a reporter gene, usually luciferase, which is introduced into target cells and expressed. Methods to generate HCV pseudoparticles (HCVpps) commonly use murine leukemia virus or HIV derived vectors [127,128]. Our group and others have utilized these systems to characterize extensive panels of patient-derived E1E2 sequences allowing important characterisation of these unique isolates [57,129,130,131]. These studies have shown that neutralisation sensitivity of patient derived isolates to both sera and monoclonal nAbs is markedly varied and not associated with genotype but is isolate dependent. Furthermore, it has been shown that the HCV reference isolate H77, a Gt1a isolate that was first adopted due to its ability to infect and cause disease in chimpanzees [132,133,134], is more susceptible to antibody-mediated neutralisation than many patient-derived isolates [102]. This finding is of significance since H77 neutralisation has been used to validate multiple vaccine candidates and therefore these data provide limited insight into how effective a candidate may be towards clinically relevant isolates. H77 has also been used in the design of immunogens with moderate success, although it could be argued that immunogen design based on more nAb-resistant isolates may elicit more potent nAbs.

The ability to generate both HCVcc and HCVpp provide essential tools for the in vitro study of HCV and assessing the neutralising breadth of sera and monoclonal nAbs. The importance of isolate panels for the validation of vaccine candidates is exemplified in the field of HIV vaccine research, in which candidates have been tested against an extensive panel of 109 isolates developed by Seaman et al. [135], which are grouped based on neutralisation susceptibility. In this way humoral responses following vaccination can be assessed in a standardized manner allowing for relevant comparisons between candidates and vaccines that induce responses capable of neutralising the most resistant isolates can be identified for further development. At the time of writing a consensus panel of isolates is yet to be widely adopted but doing so will greatly enhance our understanding of which candidates are promising and those that are not.

#### 4.3.2. In Vivo Models

Humans are the natural host of HCV, and therefore it is imperative that suitable in vivo models are used in order to test the efficacy and safety of preclinical vaccine candidates. Arguably, the most successful model has been the chimpanzee, which is permissive to HCV infection under experimental conditions. However, ethical concerns over the use of this species has led to a ban on its use in experimental research. An alternative has been the use of chimeric humanized or transgenic mouse models which have humanized livers such as Alb-uPA/SCID mice [136,137] or those engineered to express human CD81 and occludin [138,139]. There are limitations to using chimeric mouse models, most notably that they do not exhibit cirrhosis or HCC, and are technically difficult to produce [140].

In light of the limited options available for the in vivo modelling of HCV, there has been increasing interest in other members of the *Hepacivirus* genus for use as analogues. At the time of writing there are two species of particular interest, the Rodent Hepacivirus (RHV) and the Equine Hepacivirus (EqHV). RHV was initially discovered in the species *Rattus norvegicus* in 2014 [141]. Experimental infection of different lab rat breeds leads to the establishment of permanent infection with features observed during HCV infection in humans such as hepatic fibrosis, steatosis and evidence of SVC in the Holtzman rat strain [142]. There are key contrasts between RHV and HCV infections, notably the lack of IFN-γ+ CD8+ T cell responses during RHV infection [143].

EqHV was initially discovered in canines and subsequent serology testing identified equines as the natural host [144,145]. This virus is the closest relative of HCV and there are shared features of both species such as similar levels of glycosylation of the E1E2 proteins and a conserved seed site in the 5′-UTR for the liver-specific microRNA-122 (miR-122) which contributes to stability, translation, and replication of the viral RNA [146]. Seroprevalence of EqHV is in the region of 30% of surveyed horses, with approximately 3% testing positive for viral RNA [147]. This discrepancy between serology and viral RNA may be indicative of a high viral clearance rate. Despite this potential high clearance rate, EqHV acute infection may proceed to chronicity after 6 months [148], further validating the relevancy of this virus as a model for HCV.

The use of analogous Hepacivirus species will allow for the testing of vaccine strategies and the experimental challenge in the natural host of that species, a feat that cannot currently be achieved for HCV. Whilst these approaches are likely to prove highly valuable to the field of HCV vaccine research it is important to acknowledge the differences in mammalian immune systems and how this will impact on our interpretation of experimental data. For example, the *IgVH 1-69* gene with an extended CDRH3 region has previously been described as a feature of anti-HCV specific nAbs that are elicited in SVC and therefore it is logical that a vaccine candidate could be assessed based on its ability to elicit these types of nAbs. With this in mind it is worth considering how comparable the antibodies of animal model species are to those of humans. One such difference is the length of the CDRH3 regions which are typically 16 residues long [149], however these are reduced to an average of 12 to 14 residues in rodent [149] and equine species [150], respectively. The CDRH3 region can be critical for the formation of the antigen-antibody complex and extended CDRH3 loops are effective at penetrating the glycan shield of viral envelope proteins, a favorable characteristic of anti-HCV nAbs. This raises an important question about the relevance and suitability of current animal models as predictors of the human antibody response. Similarly, the paradigm of vaccine research has been to test novel candidates in small animals followed by larger animal species before clinical testing in humans. This approach enables evaluation of immunogenicity and safety prior to exposure of an immunogen in humans. However, questions should be asked as to whether this approach has hindered the progress of at least some HCV vaccine candidates because of differences between the model and human antibody repertoires.

### 4.4. Rational Design of Immunogens to Elicit Cross-Reactive Neutralising Antibodies

The informed, rational design of immunogens will be vital in order to produce a successful HCV vaccine. As advances in the analysis of antibody repertoires are allowing deeper insight into the types of nAbs that are associated with SVC, there is potential to use this information to develop immunogens that favour the production of these types of antibodies. An example of this can be seen with the E2 epitope I region or antigenic site 412 (AS412). This linear epitope is structurally flexible and when in complex with nAbs and adopts three distinct conformations. Firstly epitope I forms an extended conformation in complex with the rat nAb 3/11, a β-hairpin conformation is observed when bound to AP33 or the human nAb HCV-1, and an intermediate conformation occurs when in complex with the human nAb HC33.1 [151]. As previously discussed, HCV may escape epitope I targeting nAbs by the mechanism of glycan shift. However, this mechanism does not abrogate and instead enhances the potency of HC33.1 [152]. With this in mind, rational design of an immunogen could strive to present the epitope I region in an intermediate conformation to bias the humoral response to elicit HC33.1-like nAbs. To date this has not been investigated however, efforts have been undertaken to present epitope I in the β-hairpin conformation using cyclic peptides based on θ-defensin which adopts a similar conformation [153]. This approach induced nAb responses when tested in mice however these were lower compared to mice immunized with E2 [153]. The rational design of HCV epitopes in this way is technically challenging and is limited to targeting linear epitopes. Additionally, delivery of cyclic peptides will require further development in order to enhance the immunogenicity and stimulate greater titers of nAbs.

## 5. Vaccine Prospects

Despite the challenges faced in HCV vaccine design, there have been a variety of different vaccine approaches investigated with a small number of these candidates reaching human trials (summarized in Table 1). Currently, DNA and peptide-based vaccine candidates are actively being investigated in murine models [154,155,156,157,158]. One recently reported peptide candidate consisted of overlapping peptides derived from the p7 protein which induced antigen-specific CD4+ T cells and cytotoxic CD8+ T cells capable of targeting p7 expressing hepatocytes in vivo [154]. This study has been the first to show the immunogenicity of p7 when used as the sole target in a vaccine. Additionally, a DNA-based vaccine has been shown to induce CD4+ T cell and CD8+ T cell responses and elicit T cell memory in mice; however, a non-neutralising Ab response was also observed [156]. These vaccine candidates are in an early stage of development and therefore the following sections will discuss candidates that have been more extensively studied.

### 5.1. Recombinant Subunit Vaccines

Recombinant subunit vaccines are an attractive technology as they have previously been utilized for a variety of pathogens [159] and are economical to produce on a commercial scale. In the field of HCV vaccine research, attempts to generate a subunit vaccine have focused exclusively on targeting the HCV E proteins. The most successful candidate has been the recombinant E1E2 protein (rE1E2) derived from a Gt1a isolate. Initially, HCV-1 derived rE1E2 was used to vaccinate seven chimpanzees which resulted in a humoral response [160]. Following homologous HCV challenge, five out of seven vaccinated chimpanzees exhibited sterilising immunity with the remaining two developing acute infections that were subsequently cleared [160,161]. This was further validated in small animal models and that immunisation rE1E2 derived from a single isolate could induce cross-reactive nAbs [161]. Following this work, a phase I human trial was performed in which rE1E2 derived from the Gt1a HCV-1 isolate was administered at different doses (4 µg, 20 µg and 100 µg of rE1E2) with the MF59 adjuvant to healthy volunteers [162]. Importantly this study showed that rE1E2 can be safely tolerated in humans, a finding of significance given that a successful HCV vaccine candidate will likely require these proteins or derivatives of them. Polyfunctional CD4+ T cell responses were detected and the magnitude of this response was inversely related to dosage. Humoral responses and nAbs were initially reported [162,163] and further testing using a diverse HCVcc panel with isolates from Gt1-7 showed that 3/16 volunteers had elicited cross reactive nAbs [164]. Additionally, post vaccination sera from 5 volunteers competed with nAbs AP33, AR3B, AR4A, AR5A and IGH526, showing that the humoral response targeted both E1, E2 and the E1E2 heterodimer [165]. The ability for rE1E2 from a single isolate to induce nAbs in humans is significant and encouraging although work should be undertaken to enhance the efficacy at which nAbs are generated. A key issue with rE1E2 was purification which relied on lectin-based affinity methods that were low affinity and non-specific [166]. Furthermore, efforts to improve rE1E2 production were made by removing the transmembrane domains of the E protein. However, this truncated E1E2 was found to be a weak immunogen that elicited low levels of nAbs [167]. In light of these issues, both flag-tagged and Fc-tagged rE1E2 have been investigated and elicit nAbs in mice at comparable levels to wild type rE1E2 [166,168].

Another subunit vaccine approach has been the use of truncated soluble E2 (sE2) which results in a secreted form of the E2 ectodomain. A candidate of particular note is the H77 derived sE2384-661 construct with deleted HVR1, HVR2 and IgVR (sE2_Δ123_) which can be expressed in a correct conformation which interacts with CD81 and conformation sensitive antibodies [169,170,171]. Vietheer et al. [170] reported that the expression of sE2_Δ123_ in mammalian cells results in biochemically and immunogenically distinct isoforms of this protein that exist as monomeric, dimeric, pentameric and a high molecular weight aggregate (HMW1; estimated at 51 protomers). Immunisation of guinea pigs with different isoforms showed that HMW1 elicited greater titres of cross-reactive nAbs that could neutralise isolates from Gt2a, 4a, 5a, 6a and 7a expressed in a HVCcc system [170]. Additionally, HMW1 had exhibited a reduced activity with non-neutralising antibodies, suggesting that this aggregate focuses the humoral immune response away from these epitopes, allowing for greater focusing to nAb epitopes [170]. Whilst this initial report is promising, it was noted that sE2_Δ123_ reduced homologous neutralisation activity, a likely result of the removal of HVR1 which was also observed by Law and colleagues [172].

### 5.2. Virus-Like Particles

Virus-like particles (VLPs) are a promising area of vaccine research and have been investigated extensively to design a HCV vaccine [173]. To date, there are two widely used commercial vaccines that utilize VLP technology, the hepatitis B virus (HBV) and human papilloma virus vaccines. VLPs are formed when the structural proteins of a virus assemble in a genome-independent manner to produce a particle that resembles the native virus whilst lacking the ability to replicate and are more immunogenic than soluble protein due to their repetitive structure and ability to drain into tissue lymph nodes [107,174].

HCV-LPs were first described by Baumert et al. [175] in which VLPs were expressed in the insect cell line Sf9 following transduction with a recombinant baculovirus containing the core E1 and E2 HCV structural proteins. These HCV-LPs elicited HCV-specific IgG production, IFN-γ secreting CD8+ T cell and CD4+ T cell mediated cellular responses in mice and baboons [176,177,178]. Immunisation of four chimpanzees with four doses of HCV-LPs resulted in CD4+ and CD8+ T cell responses in all animals [179]. Subsequent challenge with homologous HCV resulted in a reduced viremia in vaccinated animals compared to controls with two animals testing negative for HCV two years post challenge [179]. Interestingly, humoral responses in chimpanzees were not detectable in three of these animals and appear to have been absent during the reduction of viremia. Further work on this candidate has not been progressed since this first study in chimpanzees. However, it is an important example for future HCV vaccine work, as it showed that viral clearance can be achieved using an experimental VLP vaccine candidate. Despite work on this candidate ceasing, HCV-LPs continued to be investigated. Recently, a HCV-LP system has been established through the transduction of the human hepatoma cell line Huh 7 with recombinant adenovirus encoding the structural genes of HCV [180]. Initially designed using the H77 isolate, further development has expanded by generating HCV-LPs using the structural sequences of different isolates representing Gt1b, 2a and 3a subtypes to produce HCV-LPs with a conformation close to that the wild type virus [180,181,182,183]. Trials of this candidate in mice showed the induction of nAbs and activation of both CD4+ and CD8+ T cell responses and this has recently been reported in a vaccinated Landrace pigs [184]. To date, assessment of the humoral response has demonstrated that this vaccine elicits nAbs with activity towards homologous strains. Heterologous neutralisation data have not been reported for this candidate. Testing the sera from animals immunized with this quadrivalent vaccine against a diverse panel of HCV isolates would be of particular interest not only for this candidate but to inform future vaccine design.

### 5.3. Viral Vector Vaccines

Viral vectors have the capacity to induce high level cellular immune responses [185], making them an attractive approach for HCV candidates. Viral vectors of particular interest are human adenovirus 6 (HuAd6), chimpanzees Ad 3 (ChAd3) and modified vaccinia ankara (MVA) [186]. The only HCV vaccine candidates to be tested in phase I and phase I/II human trials has been ChAd3 and MVA which each encoded HCV NS3, NS4a, NS4b, NS5a and inactivated NS5b (NSmut) derived from the Gt1b BK isolate [187,188]. These candidates were tested together in a heterologous primer/boost strategy in which healthy human volunteers were primed with ChAd3-NSmut and boosted with MVA-NSmut 8 weeks later [188]. The results of this phase I study showed that prime/boost ChAd3/MVA-NSmut induced both CD4+ T cell and CD8+ T cell responses directed towards epitopes on all five HCV non-structural proteins, importantly the effect of the MVA-NSmut enhanced the T cell responses and these were detectable at week 70 [188]. Given the promising cellular responses observed in the phase I trial, this approach was tested in patients with chronic HCV infections although this was unable to reverse T cell exhaustion [189], ruling out the use of ChAd3/MVA-NSmut as a therapeutic vaccine. Recently ChAd3/MVA-NSmut was tested in a large phase I/II trial involving 548 high risk individuals was concluded (clinicaltrials.gov identifier: NCT01436357). At the time of writing, this trial has concluded, and data have yet to be published. However, a press release statement from the National Institute of Allergy and Infectious Disease stated that 14 vaccinated individuals became chronically infected with HCV, suggesting that this candidate does not provide protection against HCV infection [190]. Understanding the reasons for this failure will be vital to inform our understanding of vaccine design. Given that the ChAd3/MVA-NSmut candidate exclusively targeted T-cell epitopes, the failure of this vaccine candidate could further emphasize the need for both humoral and cellular immune responses in clearance of acute infections.

## 6. Conclusions

In the 30 years since the discovery of HCV, it has been apparent that this virus is highly complex and presents a major public health challenge. Encouragingly, efforts toward a vaccine continue and investigation into a range of approaches have yielded interesting results. The vast improvements in in vitro analysis of HCV have greatly aided our understanding of how nAbs work, informing rational vaccine design. In this review, we have highlighted the potential advantages of adopting reference panels of patient-derived E1E2 sequences in order to assess vaccine candidates for their potential to induce nAbs capable of targeting circulating HCV strains. Adopting this approach will provide a more rigorous tool for evaluating the nAb response produced by novel HCV vaccine candidates a characteristic that will likely be essential in a successful HCV vaccine.

## Figures and Tables

**Table 1:** Summary of recent hepatitis C virus vaccine candidates. Subunit, virus-like particle (VLP), viral vector, peptide and DNA vaccine approaches are listed with the vaccine target, genotype (Gt) and isolate from which the candidate was derived, tested species and the humoral and cellular immune responses were reported.

Vaccine Type	HCV Target	HCV Strain	Tested Species	Antibody Response *	CD4+ T Cell Response ^†^	CD8+ T Cell Response ^†^	Ref.
**Subunit**							
HCV-1 rE1E2	E1E2	Gt1a HCV-1	humans	homologous and heterologous	yes	N.D	[162,164]
H77 sE2_Δ123_	E2core	Gt1a H77	guinea pigs	homologous and heterologous	N.D	N.D	[170]
**Virus-Like particles**							
core, E1, E2 from Gt1a, 1b, 2a and 3a	core, E1, E2	Gt1a H77, Gt1b BK, Gt2a JFH1, Gt3a	mice, pigs	homologous neutralising antibodies	yes	yes	[181,184]
HBV/HCV-LPs	E1, E2	Gt1a H77	rabbit	homologous, heterologous activity towards Gt1a and 1b, reduced activity towards Gt2a and 3a isolates.	N.D	N.D	[191]
HBV/HCV-LPs	linear E1 and E2 epitopes	not stated	mice	heterologous towards Gt1a, 1b and 2a	N.D	N.D	[192]
murine leukaemia VLP-HCVE1E2	E1, E2	Gt1a H77	mice, macaques	homologous, heterologous towards Gt1b, 2a, 2b and 4c	yes	N.D	[193]
**Viral vector**							
ChAd3/MVA-Nsmut	NS3, NS4a, NS4b, NS5a, NS5b	Gt1a BK	humans	N/A	yes	yes	[188]
**Peptide**							
p7	p7	Gt1b J4	mice	N/A	yes	yes	[154]
HCVp6-MAP	E1, E2, NS4b, NS5a, NS5b	Gt4a ED43	mice	homologous, heterologous towards JFH1	yes	yes	[155]
**DNA**							
pVax-sE1E2-IMX313P	E1, E2	Gt1b	mice	homologous, heterologous towards Gt1a, 1b, 2a, 2b, 3a, 4a, 5, 6	yes	N.D	[158]
DREP-HCV/MVA-HCV	core, E1, E1, p7, NS2, NS3	Gt1a H77	mice	non-neutralising IgG	yes	yes	[156]
pVax-N3-NS5b	NS3, NS4, NS5b	Gt1b, Gt3a	mice	N/A	yes	yes	[157]

* N/A (not applicable) for vaccine candidates that are not designed to elicit HCV-specific B cell responses. ^†^ N.D (not determined) in the study.
